# Decoding Humor Experiences from Brain Activity of People Viewing Comedy Movies

**DOI:** 10.1371/journal.pone.0081009

**Published:** 2013-12-04

**Authors:** Yasuhito Sawahata, Kazuteru Komine, Toshiya Morita, Nobuyuki Hiruma

**Affiliations:** Science and Technology Research Laboratories, NHK (Japan Broadcasting Corporation), Setagaya, Tokyo, Japan; The University of Tokyo Hospital, Japan

## Abstract

Humans naturally have a sense of humor. Experiencing humor not only encourages social interactions, but also produces positive physiological effects on the human body, such as lowering blood pressure. Recent neuro-imaging studies have shown evidence for distinct mental state changes at work in people experiencing humor. However, the temporal characteristics of these changes remain elusive. In this paper, we objectively measured humor-related mental states from single-trial functional magnetic resonance imaging (fMRI) data obtained while subjects viewed comedy TV programs. Measured fMRI data were labeled on the basis of the lag before or after the viewer’s perception of humor (humor onset) determined by the viewer-reported humor experiences during the fMRI scans. We trained multiple binary classifiers, or decoders, to distinguish between fMRI data obtained at each lag from ones obtained during a neutral state in which subjects were not experiencing humor. As a result, in the right dorsolateral prefrontal cortex and the right temporal area, the decoders showed significant classification accuracies even at two seconds ahead of the humor onsets. Furthermore, given a time series of fMRI data obtained during movie viewing, we found that the decoders with significant performance were also able to predict the upcoming humor events on a volume-by-volume basis. Taking into account the hemodynamic delay, our results suggest that the upcoming humor events are encoded in specific brain areas up to about five seconds before the awareness of experiencing humor. Our results provide evidence that there exists a mental state lasting for a few seconds before actual humor perception, as if a viewer is expecting the future humorous events.

## Introduction

A sense of humor is a common human characteristic that people in many cultures experience. Humor not only encourages social interaction, but also produces positive effects for the human body, such as healthy physiological changes [1]. Comedians and movie directors work hard to stimulate humor in audiences. Would a better understanding of the mechanisms of humor perception help comedians and movie directors better amuse audiences? Previous psychological studies have suggested that there are mental stages associated with experiencing humor [2], [3]. Furthermore, recent neuro-imaging studies have revealed physiological evidence for a relationship between mental stages and experiencing humor [4]–[18]. For example, Chan et al. examined fMRI activity while audiences listened to several humorous short stories, and showed that humor-related mental states correlated with activity in distinct brain areas; incongruity detection, resolution and elaboration of humor were respectively involved in the right middle temporal gyrus and right medial frontal gyrus, the left superior frontal gyrus and left inferior parietal lobule, and the left ventromedial prefrontal cortex, the bilateral parahippocampal gyri and the bilateral amygdale [16], [18]. Such physiological measurements for experiencing humor should be of value to the creators of comedy shows and movies, because it would help them to know the detailed reactions of audiences and the objective value of their products.

It is a challenging task to objectively measure the dynamic mental events underlying such subjective experiences during movie watching. With advances in neuro-imaging technologies, a great number of studies [4]–[18] have successfully measured brain activity related to the perception of humor. However, these studies did not focus on a single humorous event but on multiple humorous events within intentionally designed experiments, as they sought only to the distinguish brain activity in the humorous trials from that in the non-humorous trials. If it is possible to monitor the viewer’s mental states on a volume-by-volume basis of fMRI images, we can expect that the results will not only be of value to the creators of comedies but also improve our understanding of the dynamic processes in the brain related to humor.

To predict humor-related mental events, we analyzed fMRI data using a decoding approach [19]–[22] that classifies given brain activity patterns into pre-defined brain states by using a statistical machine learning algorithm. A general linear model (GLM)-based approach [23], which is often used in neuro-imaging studies about humor, is not suitable for mental event prediction, since it is an encoding model that predicts brain activity from stimuli, experimental or task variables [24]; the modeling direction is opposite from ours. By using a decoding approach, once a mapping from brain activity patterns to humor-related mental states is learned, the mental states of viewers can be predicted from individual fMRI volumes taken while they are watching comedic situations. It is reasonable to take the decoding approach to measure the subjective mental state changes that cannot be externally observed.

Recent neuro-imaging studies [16], [18] have shown that the neural correlates of humor processing have two stages: comprehension and elaboration. These studies suggested that there should be humor-related mental state changes before and after humorous events. However, the time scale of the dynamic humor processing remains elusive. Since a mental state can be predicted from single-trial brain activity data by using the decoding approach, we can expect that the characteristics of the mental state changes can be extracted with the same temporal resolution as that of the brain activity data.

In the present study, we investigated the dynamic mental state changes by applying the decoding approach to single-trial fMRI data obtained while subjects viewed comedy TV programs. We hypothesized that each single-trial observation data observed during humor experiences may have distinct information from that observed during ‘neutral’ states in which a viewer did not experience humor. We conducted an fMRI experiment in which a viewer watched comedy TV programs in an fMRI scanner and reported the humor levels that he or she experienced. Regions of interest (ROIs) were defined in each subject by dividing the whole brain into sub-regions based on anatomical landmarks. We constructed multiple decoders for each ROI at each lag before and after the onset of a humor report in order to investigate whether brain activity patterns obtained before and/or after the humor perception and during neutral states can be classified on a trial-by-trial basis. By testing the performance of each trained decoder, we could predict where in the brain and when the humor-related information would be elicited while viewing comedy TV shows.

## Materials and Methods

### Subjects

Ten healthy adults (mean age 28.2±7.99 years, range 20–44; 7 men and 3 women) participated in the movie-viewing experiment. Ten healthy adults (Four of them also participated in the movie-viewing experiment; mean age 27.7±6.77 years, range 20–44; 8 men and 2 women) participated in the control experiment. All the subjects were native Japanese speakers, and had no history of psychiatric or neurological disorders. The subjects gave written informed consent and the study was approved by the Ethics Committee of the NHK Science and Technology Research Laboratories.

### Stimuli

Ten stand-up comedies, or *manzai* in Japanese, in which small groups of comedians performed funny dialogues were selected from comedy TV shows, called ‘*On-air Battle*’, broadcast on NHK TV in Japan. Laughter of audiences in the recording hall was also included in the movies. Each movie was about 4.5 minutes long. Visual stimuli were presented within 10°×10° visual angles, rear-projected onto a screen placed in the scanner bore using an LCD projector and viewed via a mirror mounted on the head coil. Audio stimuli were given by MRI compatible head-phones.

### Design and Tasks

Movie-viewing experiment. In each run, a movie was presented between a 32 s initial rest period and a 30 s closing rest period in which a fixation cross on a gray background was shown at the center of the screen. Hence, each run had about 5.5 minutes of stimulus presentation in total. During the rest periods, subjects fixated on the cross. During the movie-viewing periods, subjects viewed stimuli (the standup comedies) without fixation and reported the magnitude of humor they were experiencing by manipulating a slider-style response device (fORP; Cambridge Research Systems, Rochester, Kent, UK). Subjects were instructed not to move their head even if they wanted to laugh.

Control experiment. To investigate whether decoding results were derived from humor processing or manipulations of the response device, we conducted control experiments in which subjects manipulated the response device without viewing the comedy movies. Subjects were instructed to manipulate the slider device so as to mimic the motion of a cartoon slider tab shown in a screen. The control experiment involved a 32 s initial rest block followed by a 16 s manipulation block and a 16 s rest block repeated six times per run. In total, eight runs were conducted. In each manipulation block, the cartoon slider was moved periodically at 0.5 Hz.

### Response data preprocessing

**Figure 1:**
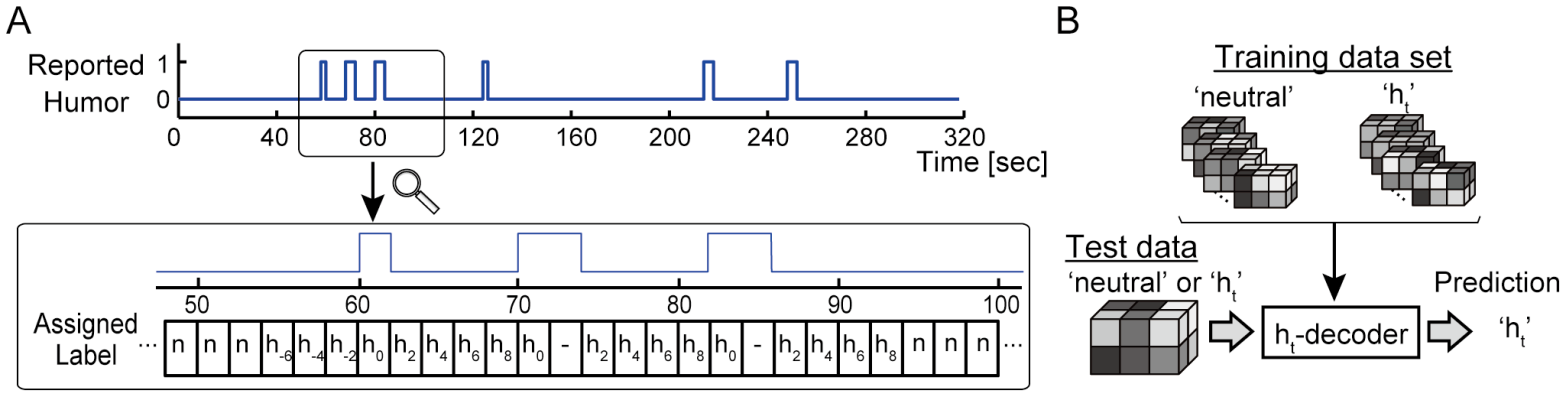
Decoding of humor experiences. (A) Definition of neutral and humor labels. Neutral and eight types of humor labels are defined based on the lag before or after the onset of a humor report. The upper plot shows a time-series of humor reports. The lower plot is the magnified view of the highlighted area shown on the upper plot, showing examples of the defined labels. Eight distinct humor labels ‘h*_t_*’ (*t*  =  -6–8 for each 2 s) are assigned to fMRI time-series data from six seconds before to eight seconds after the onset of each humor report. The other fMRI data that are more than six seconds before or more than eight seconds after the humor onset are assigned the ‘neutral’ label. Note that h*_t_* with *t* > 0 is given priority over h*_t_* with *t*<0 when multiple humor reports occur in a short period. When consecutive ‘h_0_’ labels could be assigned in the case that a subject kept a slider up for several seconds, only the first ‘h_0_’ label is placed and no labels are assigned to fMRI data that would otherwise be assigned the successive ‘h_0_’ labels. (B) Training and testing humor decoders. For each humor label h*_t_*, we constructed decoders that predict ‘h*_t_*‘ or ‘neutral’ for a given fMRI data sample. A training and testing data set for the h*_t_*-decoder were created by choosing h*_t_*- and neutral-labeled data samples from all the data obtained during movie viewing. Note that the training and testing data sets were independent, as nine out of ten runs were used for training and the remaining one run was used for creating the testing data set.

Responses from a slider device were converted into labels for fMRI data based on the lag before or after a humor onset (Figure 1). First, measured response values (sampled with 100 Hz) were binarized into two clusters, “presence” or “non-presence” of slider manipulation, by applying *k*-means clustering with *k* = 2 to the response values of all runs. Next, to associate response data with the simultaneously observed fMRI images, we sorted the binarized responses into 2 s long bins and regarded a bin with at least one slider manipulation as a slider-manipulated bin. The slider-manipulated bins were thus defined as humor onsets and assigned ‘h_0_’ labels (0 s to the humor onset). Subsequently, time points that were *t* s to the nearest humor onset were assigned ‘h*_t_*’ labels (*t* s to the humor onset; -6 ≤ *t* ≤ 8 for each 2 s). ‘Neutral’ labels were assigned to bins that were temporally far away from the humor onset (more than 6 s before or 8 s after the humor onset). Note that h*_t_* with *t* > 0 is given priority over h*_t_* with *t*<0 when the labeling criteria indicated that multiple labels could be assigned to a bin. Furthermore, in the cases that consecutive ‘h_0_’ labels could be assigned to data samples by the above labeling criteria when subjects kept the slider up for several seconds, only the first ‘h_0_’ label was assigned and no labels were assigned to the samples that were supposed to be assigned the subsequent ‘h_0_’ labels. Hence, the fMRI data associated with h*_t_* labels (*t* ≤ 0) were always the ones obtained before or simultaneously with the humor onsets.

### Monitoring changes in facial expression

To take into account artifacts caused by facial expressions or head motions, four light-reflective markers were attached around the subject’s mouth (both corners of the mouth and edges of the lower lip). By using an infrared camera (SONY HDR-CX700V; used in the night shot mode), marker motions were recorded with 1920×1080 pixels at 60 frames per second from the side of the scanner-bed through the mirror mounted on the head-coil (i.e. two mirrors were mounted on the head-coil: one for stimulus-viewing and another for marker motion recording).

We created the alternative labels from the time series of the marker positions. First, each coordinate value of markers was normalized relative to the averaged coordinate value over a run. Next, the relative values of the marker positions were converted into one-dimensional signals by computing their squared sum at each time point. Then, the signals underwent linear trend removal within each run. The motion-based labels were extracted using the same procedure described in the response data preprocessing, but the response data were substituted with the computed time series data of the marker positions.

### Extracting sounds of audience laughter contained in stimuli

To determine whether the decoding results were derived from the humor process in the brain or sensory cues in funny scenes regardless of the subject’s humor sensation, we extracted the time courses of sounds of laughter independently of other sounds such as the speech of the comedians. For this, we recruited two volunteers, who had not participated in the fMRI experiments, and instructed them to manipulate a slider device in response to the loudness of the laughter contained in stimulus movies while viewing them. Then, by using the average of the obtained laughter sound presences, we created laughter-based labels by using the same procedure as in the response data preprocessing.

### MRI acquisition

MRI data were all obtained using a 3T MRI scanner (MAGNETOM Trio A Tim; Siemens, Erlangen, Germany) using a standard head coil at the ATR Brain Activity Imaging Center (Kyoto, Japan). An interleaved T2*-weighted gradient-echo planar imaging (EPI) scan was performed to acquire functional images to cover the entire brain (TR, 2000 ms; TE, 30 ms; flip angle, 80°; FOV, 224×224 mm; voxel size, 3.5×3.5×4; slice gap, 1 mm; number of slices, 30). T2-weighted turbo spin echo images were scanned to acquire high-resolution anatomical images of the same slices used for the EPI (TR, 6000 ms; TE, 57 ms; flip angle, 90°;FOV, 224×224 mm; voxel size, 0.875×0.875×4.0 mm). T1-weighted magnetization-prepared rapid-acquisition gradient echo (MP-RAGE) fine-structural images of the whole head were also acquired (TR, 2250 ms; TE, 3.06 ms; TI, 900 ms; flip angle, 9°; FOV, 256×256 mm; voxel size, 1.0×1.0×1.0 mm).

### MRI data preprocessing

The first 2 s scans of each run were discarded to avoid the effect of the instability of the MRI scanner. Note that our discarding 2 s of the scan at the beginning was enough since the MRI scanner had a function to avoid instability in the scans; two volumes (4 s scans with TR = 2 s) are discarded internally before outputting the EPI files. The acquired fMRI data underwent slice-timing correction and three-dimensional motion correction by SPM5 (http://www.fil.ion.ucl.ac.uk/spm). The data were then coregistered to the within-session high-resolution anatomical image of the same slices used for EPI and subsequently to the whole-head high-resolution anatomical image. The coregistered data were reinterpolated as 3.5×3.5×5 mm voxels.

**Figure 2:**
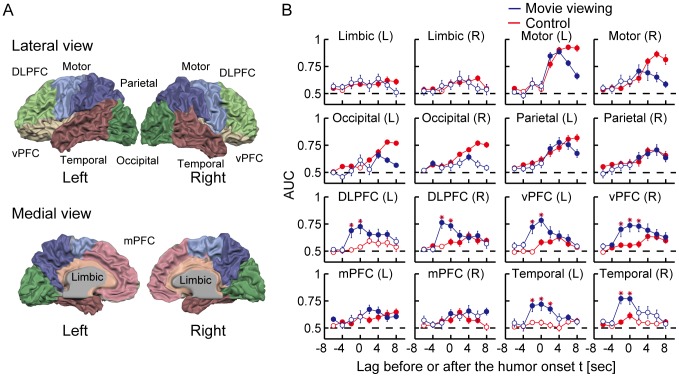
Definition of ROIs and performance of decoding humor experiences versus that of response device manipulation. (A) Regions of interest (ROIs) were defined in each subject by dividing the whole brain into 16 sub-regions (eight sub-regions in each hemisphere) based on anatomical landmarks. The upper and lower brain images depict examples of the color-coded ROIs for S1 shown in the lateral and medial views of the left and right hemispheres. (B) Each graph depicts the decoding performances against the lag before and after the humor-onset obtained from each anatomically defined brain area. The blue lines correspond to the decoding results for the movie-viewing experiment, whereas the red lines correspond to the results of the control experiment in which subjects manipulated a response device following instructions shown on the screen instead of viewing comedy movies. The filled and open circles on the blue or red lines respectively indicate that the accuracy is significantly higher than the chance level (AUC = 0.5; a dashed line in each graph) or not significant relative to the chance level (*p*<0.05, *t*-test, false discovery rate [FDR] corrected for multiple comparisons). A red asterisk indicates a significant difference between the decoding accuracies from the movie-viewing experiment and the control experiment (*p*<0.05, *t*-test, FDR corrected for multiple comparisons). Error bars correspond to s.e.m.

The voxels used for the decoding analysis were sorted according to the anatomically selected regions of interest (ROIs), i.e., the bilateral dorsolateral prefrontal cortex (DLPFC), ventral prefrontal cortices (vPFC), medial prefrontal cortices (mPFC), temporal cortices, parietal cortices, occipital cortices, motor areas (consisting of primary motor cortices, premotor cortices and supplementary motor areas), and limbic systems (see Figure 2A). Note that our definition of DLPFC covered the lateral and dorsal side of the prefrontal cortex and was broader than the restricted definition of DLPFC, which covers only around BA9 and 46. The anatomical ROIs were defined manually using Brain Voyager QX software (Brain Innovation, Maastricht, the Netherlands); the voxel coordinates in the Talairach space around the gray-white matter boundary in the manually segmented ROIs in each individual brain were transformed into the coordinates of the EPI images. The average numbers of voxels across subjects in each ROI were shown in Table 1.

**Table 1:** Number of voxels in the anatomically defined ROIs.

Region of Interest	Left hemisphere	Right hemisphere
DLPFC	1101±95.9	1141±128.6
vPFC	555.0±127.7	569.8±126.0
mPFC	601.4±109.6	564.8±222.0
Temporal	1710.0±213.9	1755.0±177.1
Parietal	1022.9±158.0	1037.3±128.0
Occpital	1383.1±156.9	1472.7±259.9
Motor	751.5±113.0	778.2±189.8
Limbic	421.6±88.5	363.7±54.67

(mean ± s.d. across subjects).

### Decoding analysis

Data samples for the decoding analysis were created by normalizing the response amplitudes of individual voxels relative to the mean amplitude of the first 30 s rest period in each run to minimize the baseline difference across runs. Labels about the lag before or after a humor onset (h_-6_ to h_8_ and neutral) were assigned to each data sample.

To examine which mental states described by the ‘lag before or after a humor onset’ can be extracted from brain activity, we trained and tested multiple decoders constructed for each lag. The decoders performed binary classifications that classified h*_t_*-labeled data from neutral-labeled data. As in conventional decoding studies [20], [21], we used the linear support vector machine as an implementation of a decoder (using the libsvm package [25]). For each h*_t_* decoder, we created a training dataset consisting of h*_t_* and neutral-labeled data samples. Since humor-reported periods tended to be short compared to the entire duration of a movie and the ratio between the number of h*_t_* and neutral-labeled data samples in a training dataset was very biased, we equalized the number of samples with both labels by omitting randomly chosen samples with neutral labels. Although we could have created a training dataset without equalizing the sample sizes, we chose to equalize them because our preliminary analysis showed that equalized datasets performed better in terms of prediction accuracy and processing time. Taking into account the variability of the training dataset created by the random sampling, we obtained a decoder in three steps. First, we created 500 different training datasets by random sampling. Next, we trained multiple decoders independently for each training dataset. Then, we determined a single decoder by averaging the weight parameters of the trained multiple decoders. To evaluate the prediction accuracy of each h*_t_* decoder, we created a test dataset consisting of h*_t_* and neutral-labeled samples. It should be noted that the label information was only used to create a test dataset and it was hidden from the decoders. To evaluate the decoding accuracy using a test dataset with a biased number of samples in each class, we computed the receiver operating characteristic (ROC) curve on the basis of decision values for test samples and obtained the area under the ROC curve (AUC), which represents the performance of a pattern classifier by a value between 0 and 1 (chance level, AUC = 0.5; perfect classification, AUC = 1.0). The decoding accuracy was computed in a cross validation manner whereby data samples in one run were used to test a decoder trained with the data samples from all other runs, and this training-test set was repeated for all runs.

Subsequently, to determine whether the decoders could predict the viewer’s mental state even from the observed time-series fMRI data, all data samples in a test run were given to the decoders that showed above chance-level performance in the former analysis. Predictions were made in a similar way to the cross-validation manner described above; data samples in one run were input into a decoder trained with the data samples from all the other runs, and this training-prediction set was repeated for all combinations.

In a separate analysis to determine whether the decoding accuracies were from humor processes in the brain or from other artifacts, such as facial expression changes or head motion elicited by laughing, we conducted decoding analyses with the alternative labels extracted from facial marker motions instead of the original labels extracted from the humor reports of the subjects. Finally, the prediction accuracies of the original decoding and the decoding using marker motion were compared.

Furthermore, we conducted an additional decoding analysis with labels identifying audience laughter in the movie stimuli to see if we could determine whether the original decoding results were derived from the subjective humor process or just from specific visual or auditory cues embedded in funny scenes independent of individual humor sensations. The prediction accuracies of the original decoding and the decoding with the laughter sound-based labels were compared in the same way as the decoding using facial marker motion explained above.

## Results

### Behavioral results

Subjects reported their humor experiences using a slider device. According to the interviews after the fMRI scans, all the subjects said they were able to respond without a large delay in their awareness of humor. The number of humor reports for each movie is shown in Table 2. We labeled data samples on the basis of the humor reports. The number of data samples for each label across the subjects is shown in Table 3.

**Table 2:** Number of humor reports for each movie.

Movie ID	Number of humor reports
1	3.20±2.82
2	2.30±1.82
3	2.20±1.75
4	3.50±4.37
5	4.00±4.32
6	2.90±3.03
7	3.50±4.00
8	3.40±3.37
9	5.10±3.63
10	2.80±2.86

(mean ± s.d. across subjects).

**Table 3:** Number of data samples for each label.

Label	Number of data samples
h_-6_	21.8±12.8
h_-4_	23.3±14.4
h_-2_	24.6±15.7
h_0_	32.9±25.9
h_2_	32.9±25.9
h_4_	31.8±24.1
h_6_	29.9±21.8
h_8_	28.8±20.2
neutral	1038.1±173.3

(mean ± s.d. across subjects).

### Decoding results

We first trained the h*_t_*-decoders using voxels from the anatomically selected ROIs and validated the performance of each decoder. Compared with the decoding accuracies obtained from the control experiment, we found humor-specific information was encoded two seconds before or at the same time as a humor onset in the brain regions including bilateral DLPFC, vPFC and temporal areas (Figure 2). Although significant prediction accuracies above the chance level occurred two or more seconds after humor reports in the brain regions including bilateral motor and parietal areas, these results could not reject the possibility of the relevance of the motor manipulation, since significant performances were also found in the control experiment. Although we found the bilateral motor activation induced by a motor action of the right hand, it is likely because the laterality is not always maintained, as reported by [26]. Also, although significant prediction accuracies were found in the occipital areas, we could not reject the possibility that they were derived from the differences in the visual stimuli under the h*_t_* and neutral conditions and not from subjective states related to humor processing, because we did not control the content of the original movie stimuli. Hence, taking into account the hemodynamic delay of the BOLD responses, which was about three seconds after the neural activity, the predictive neural process in broad areas in the PFC and temporal areas should be preceded three or five seconds by the awareness of experiencing humor.

**Figure 3:**
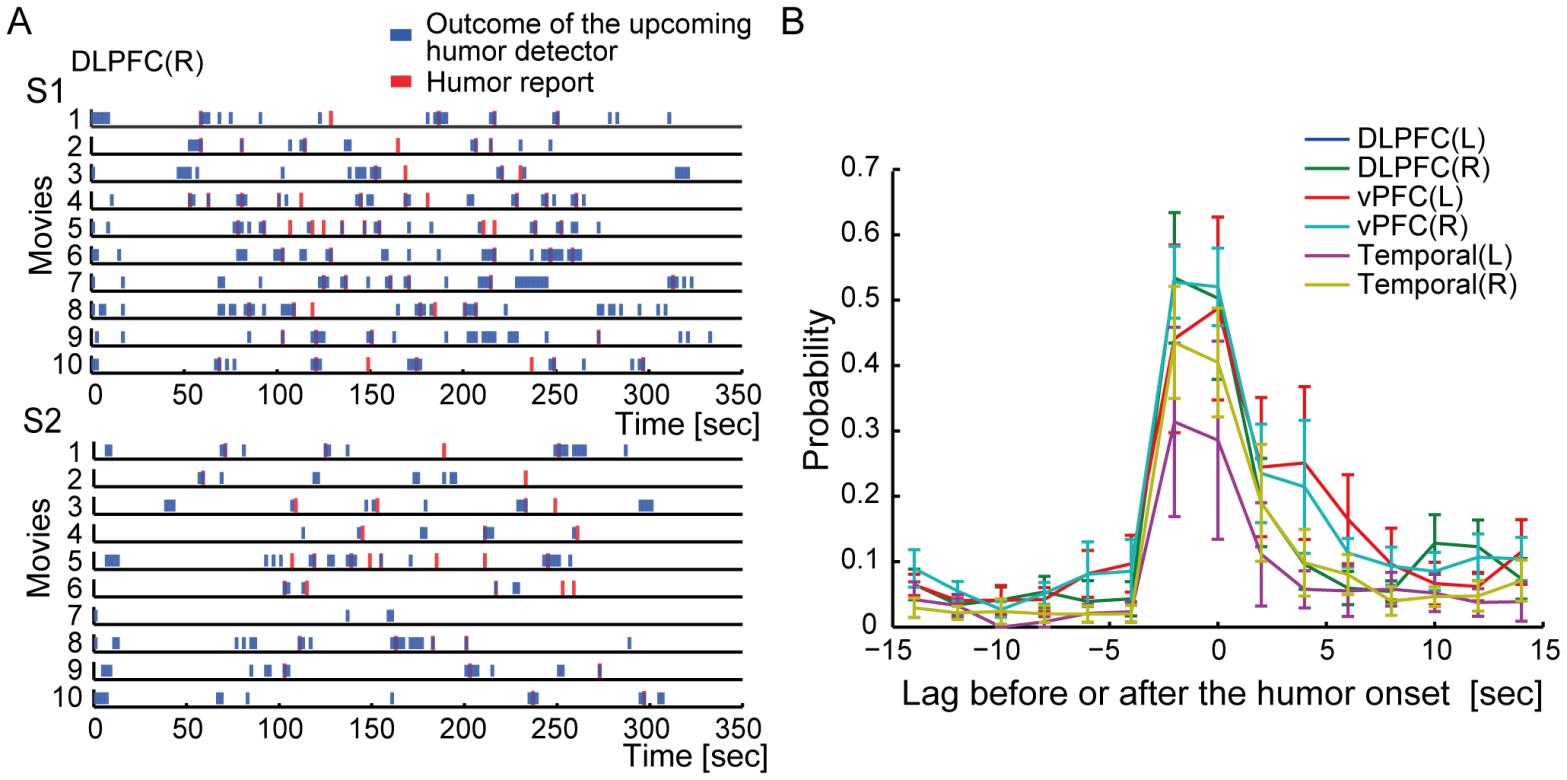
Prediction of upcoming humor experiences. (A) Temporal relationship between the predicted upcoming humor and the actual humor reports. The fMRI activity patterns in the right DLPFC obtained at each time point during movie-viewing were given to the h_-2_-decoder (upcoming humor detector) to predict the upcoming humorous events. The blue and red rasters indicate the outcome of the upcoming humor detector and the time point of the reported humor. Prediction results for all movie stimuli are depicted from the top to bottom in each panel (the upper and lower panels are for S1 and S2, respectively). (B) Detection probability of the upcoming humor experiences across the lag to the onset of humor reports. The colored solid lines correspond to the results for the upcoming humor detector constructed for the brain areas that showed the significant decoding accuracy at *t* = -2 s (shown in Figure 2). Error bars correspond to s.e.m.

Figure 3 illustrates the decoding outcomes of the h_-2_-decoder for the sequences of single volume fMRI data in each run. Figure 3A illustrates the outcomes of the h_-2_-decoder, denoted by upcoming humor detector, for the right DLPFC of S1 and S2. Since we chose the h_-2_-decoder that showed significant decoding accuracy using fMRI data obtained two seconds before the humor onset, the high detection probability of the upcoming humor at the same lag is not a surprising result. However, the results show that the positive outcomes of the h_-2_-decoder were found not only two seconds prior to the humor onsets but also at the subsequent time points. By sorting the predictions of upcoming humor relative to the humor onsets, we found that the h_-2_-decoders constructed for the bilateral DLPFC, vPFC, and temporal areas predicted the upcoming humor not only two seconds before the humor onsets but also at the same time as them (Figure 3B). A similar tendency was also observed from the h_0_-decoders that showed the significant accuracies in Figure 2B. Hence, the information extracted by the h_-2_-decoders was the same as that extracted by the h_0_-decoders. Therefore, it is likely that the fMRI activity elicited at two seconds before and at the same time as the humor onset represented the same information that anticipates the upcoming humor events.

**Figure 4:**
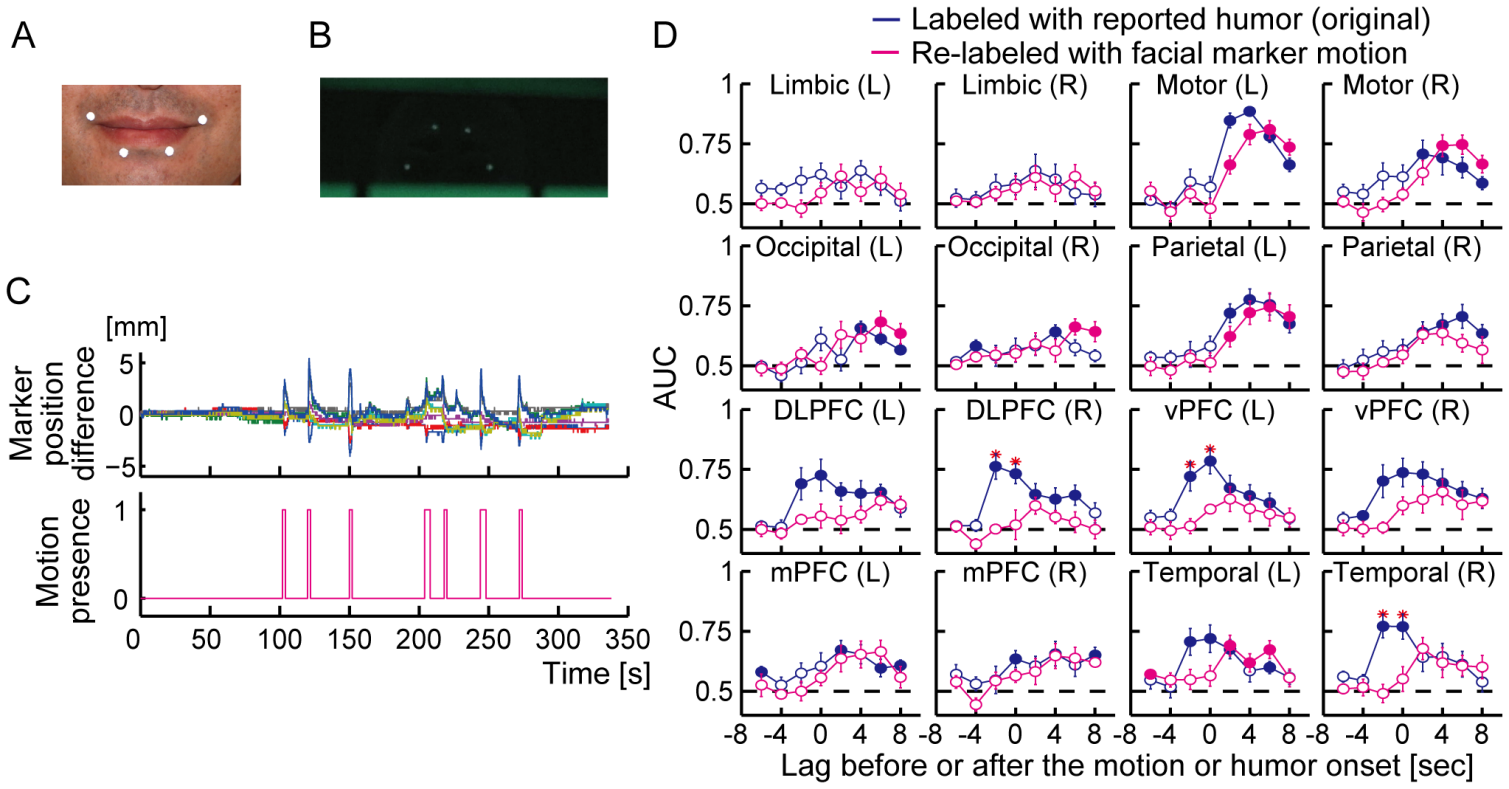
Facial skin motion did not explain the performance of the upcoming humor detectors. (A) Four light-reflective markers put around the mouth. (B) An image of the markers taken by an infrared video camera through a mirror mounted on the head coil. (C) Temporal changes in the marker positions and the determined motion labels. In the upper panel, each coordinate value of the markers is normalized relative to the averaged value over a run and plotted against time. The lower panel depicts the determined motion labels corresponding to the large facial marker motion shown in the upper panel. (D) Performance of decoding humor experiences versus that of facial marker motion. The fMRI activity patterns in each brain area were re-analyzed by labeling them with detected facial marker motion whereas the original activity patterns were labeled with humor reports. Each graph depicts the decoding performances obtained from each anatomically defined brain area across the latency to the humor or motion onset. The magenta and blue solid lines correspond to the results of the decoding of facial marker motion and the humor experiences, respectively. The filled and open circles on the magenta or blue lines respectively indicate that the accuracy is significantly higher than chance level (AUC = 0.5; a dashed line in each graph) or not significant relative to the chance level (*p*<0.05, *t*-test, FDR corrected for multiple comparisons). A red asterisk indicates a significant difference between the accuracies of the original decoding and the decoding with facial marker motion (*p*<0.05,*t*-test, FDR corrected for multiple comparisons). Error bars correspond to s.e.m.

To validate that the decoding results were not derived from artifacts, such as facial expression changes or head motions, we reanalyzed fMRI data by assigning the alternative labels extracted from facial motion changes instead of the humor reports to the data samples (see Materials and Methods). Humor onsets were determined on the basis of large changes in the facial expression whereas they were determined on the basis of humor reports given by a response device in the original analysis. The number of data samples for each facial motion based label across the subjects is shown in Table 4. The original decoding results would seem to be derived from facial motions if the decoding accuracies using the facial motion-based labels remained the same as or outperformed the original decoding accuracies. However, the decoding accuracies using the facial motion-based labels dropped at most lags in comparison with the original decoding accuracies (Figure 4). In particular, during the lags two seconds prior to the onset, the decoding accuracies using the alternative labels dropped to the chance level in the regions where the original decoding had shown significant accuracies.

**Table 4:** Number of data samples for facial marker based labels.

Label	Number of data samples
h_-6_	29.1±14.1
h_-4_	31.5±15.4
h_-2_	33.9±17.3
h_0_	48.1±28.6
h_2_	48.1±28.6
h_4_	45.5±27.2
h_6_	41.8±24.0
h_8_	39.9±22.5
neutral	901.0±203.9

(mean ± s.d. across subjects).

**Figure 5:**
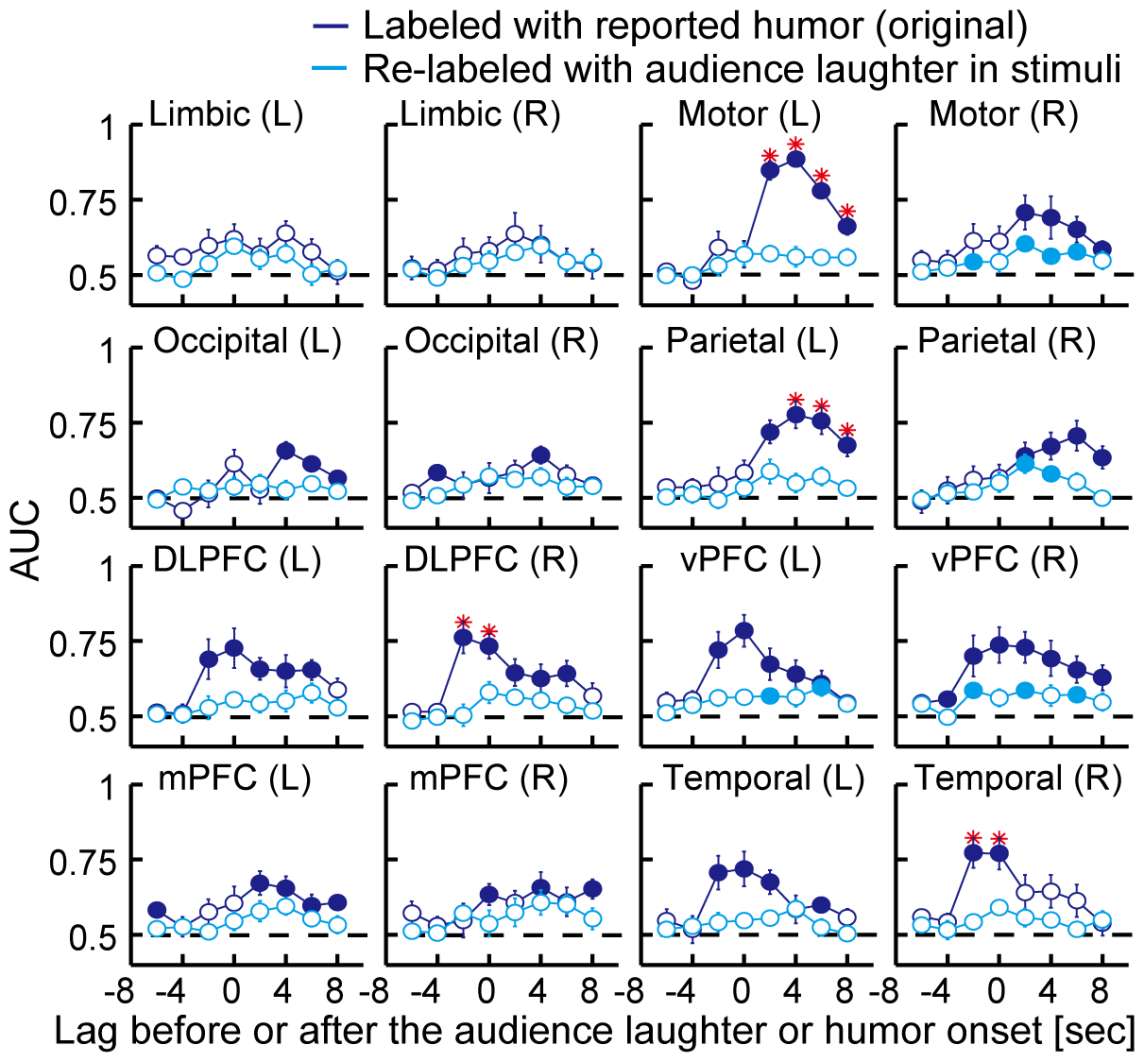
Audience laughter did not explain the performance of the upcoming humor detectors. The performance of decoding humor experiences versus that of laughter from the audience is shown. The fMRI activity patterns in each brain area were re-analyzed by labeling them with presence of sounds of laughter from the audience contained in movie stimuli whereas the original activity patterns were labeled with humor reports. Each graph depicts the decoding performances obtained from each anatomically defined brain area across the latency to the humor or motion onset. The cyan and blue solid lines correspond to the results of the decoding of laughter sounds and the humor experiences, respectively. The filled and open circles on the cyan or blue lines respectively indicate that the accuracy is significantly higher than chance level (AUC = 0.5; a dashed line in each graph) or not significant relative to the chance level (*p*<0.05, *t*-test, FDR corrected for multiple comparisons). A red asterisk indicates a significant difference between the accuracies of the original decoding and the decoding with laughter sounds (*p*<0.05, *t*-test, FDR corrected for multiple comparisons). Error bars correspond to s.e.m.

Figure 5 compares the results of the original decoding and the decoding using the laughter-based labels. The number of data samples for the laughter-based labels is shown in Table 5. The original decoding results would seem to be derived from sensory cues in funny scenes independent of humor processing if the decoding accuracies using the laughter-based labels remained the same as or outperformed the original decoding accuracies. However, the decoding accuracies using the laughter -based labels dropped at most lags, including ones before humor onsets, in comparison with the original decoding accuracies.

**Table 5:** Number of data samples for laughter-based labels.

Label	Number of data samples
h_-6_	40
h_-4_	46
h_-2_	62
h_0_	151
h_2_	151
h_4_	127
h_6_	111
h_8_	89
neutral	414

## Discussion

We have shown that the upcoming humor experiences can be decoded from fMRI responses two seconds ahead of and at the same time as humor reports in the areas including the bilateral DLPFC, vPFC, and temporal areas. The poor decoding performance during the period before the response device manipulations in the control experiments suggests that the accurate decoding of upcoming humor was not derived from motor preparation. Moreover, the changes in facial expressions and laughter from the audience in the movie stimuli cannot account for the accurate decoding, particularly in the right DLPFC and the right temporal area. Therefore, it is unlikely that the above chance level decoding of the upcoming humor, prior to the awareness of humor, from the ROIs were artifacts caused by changes in facial expression and/or sensory cues existing in funny scenes. These results suggest that a specific subjective state predictive of upcoming humor experiences exists and that it can be detected from single trial fMRI data obtained during natural movie viewing. Taking into account the hemodynamic delay, we found that the state predictive of upcoming humor experiences begins from more than two seconds before and lasts until the awareness of humor.

It is known that decision making about a motor action induces a negative brain potential in the SMA, the so-called ‘motor readiness potential,’ [27] before an actual action. However, our results that predicted upcoming humor reports are unlikely to have a connection with motor decision making, because the time scale of the activity involved in motor decision making is much shorter than the upcoming-humor prediction; a few milliseconds precedes the actual action in the decision making whereas a few seconds preceded the upcoming humor reports in our results. It should be difficult to obtain the readiness potential-related activity by using a measurement device with poor temporal resolution such as fMRI.

In our experiment, ten subjects participated in each movie-viewing and control experiment, but four individuals participated in both experiments (i.e. 16 individuals participated in total). Hence, the subjects could be categorized strictly into three groups: subjects who participated only in the movie-viewing experiments, those who participated in the control experiments, and those who participated in both. Analyses under this categorization would be appropriate if participation in a movie-viewing experiment affects the results of a control experiment. However, since the task that the subjects conducted in the control experiment was just a simple motor task, we considered that it was valid to assume there were no cross interactions between the two experiments. In fact, there were no significant differences between the mean decoding accuracies of the subjects who participated only in the control experiments and those who participated in both experiments. Thus, even with the original categorization of subjects, we believe that we could reliably determine whether the decoding accuracies were derived by humor experiences or response device manipulations.

Previous fMRI studies on humor processing [4], [9], [14]–[16], [18] have been based mostly on the incongruity resolution theory [2] or its related theories [3], in which humor processing can be divided into two or more stages, such as incongruity detection, incongruity resolution, humor appreciation, etc. These studies divided humorous episodes into two phases, a setup line and a punch line, and compared BOLD responses for punch lines with ones for various baseline conditions to examine the neural basis of incongruity detection and resolution [4], [15], [16], [18]. There is a study [14] that compared the responses to unfunny and funny episodes, without dividing them into setup and punch lines. However, the present study found a humor-related brain representation lasting for a few seconds in the setup phase. Since incongruity detection and incongruity resolution processes could be done in a moment at the end of a setup line, it is unlikely that the evidence of the present study fits incongruity theory. Indeed, the humor processing identified in the present study appears to be done before the incongruity processes. Thus, the present results suggest that there are periods in which a subject is preparing for or expecting humor events before they perceive them.

The present study found that the broad brain areas were involved in the prediction of upcoming humor perception. Particularly, brain areas, such as prefrontal and temporal areas, were shown to be involved mainly in the prediction of upcoming humor perception. The inferior frontal areas have previously been shown to be involved in understanding or inferring others’ mental states [28] and understanding semantic context in speech [29], [30]. The temporal poles and superior temporal sulcus (STS) included in the temporal ROIs have been shown to be involved in the inference of others’ intentions and recalling socially relevant memories [28], [31], [32]. These basic characteristics in the prefrontal and temporal areas may support our finding that there is mental processing for preparing or expecting upcoming humor before the perception of it.

Neuro-imaging studies of humor have indicated that the temporoparietal junction (TPJ) plays an important role in understanding jokes [5], [7], [10], [12], [13], [17]. In our analysis, areas corresponding to the TPJ were included in the parietal ROIs. Although our results cannot be used to prove the unique involvement of the TPJ in humor processing since data with information relevant to motor processing was also extracted, the parietal area indeed showed the significant decoding performance after the humor onset that was consistent with the previous studies. Further efforts, e.g., obtaining subjective reports from people viewing movies without contaminating humor processing, are needed to clarify the involvement of the TPJ in humor processing of natural and dynamic humor stimuli.

In the decoding analyses, successful decoding of upcoming humor was performed and the mean values of AUC across subjects were more than 0.7 but did not reach 0.8, which is considered to be a threshold of good accuracy. Although higher accuracies would make the results more reliable, a sufficient value of the prediction accuracy should be determined on the basis of the purpose of study and/or the requirements of the applications. Our results showed significantly higher accuracies than chance level and than in the control experiments. They at least suggest that there exists meaningful information related to upcoming humor in the fMRI activity patterns. Development of more sophisticated decoding algorithms would improve the prediction performance.

Although our method could predict upcoming humor experiences from single-trial fMRI data, it has a limitation when it comes to identifying precise brain regions involved in the mental processing. We roughly defined ROIs based on the anatomical landmarks of the brain and used a statistical learning algorithm to decode information from each ROI. This method can identify informative ROIs but needs more work to identify informative sub-regions in a ROI. In a linear classifier, since a classification is performed by thresholding a weighted sum of voxel activities, voxels with bigger absolute weight values could be interpreted as relatively informative voxels in a ROI. Although we examined the biased distribution of informative voxels shared across subjects by mapping weight values onto the surface of the normal brain, we could not find a significant tendency. The searchlight decoding method [33], in which a decoding is applied to small spherical ROIs centered at each location in the brain, could be used, but we conducted ROI-based decoding analyses because searchlight decoding is very computationally expensive. Since our framework involves iteratively choosing samples in a random fashion for creating training datasets, searchlight decoding would not be able to finish all the analyses within a realistic time frame. An algorithm that automatically selects the relevant voxels for decoding from many voxels [34] would also not be feasible because it has similar computational issues. Future improvements to these methods and/or new computational technologies may be able to overcome this limitation.

Finally, our results on predicting upcoming humor, or anticipating humorous events, suggest that it is important to make a viewer expect a humorous event and then give him or her a punch line within a few seconds to induce laughter efficiently. It would make sense that, for example, if a stern professor suddenly says a joke in a serious lecture, no student would be able to follow the joke. Of course, since our experiments did not cover all kinds of humor, further investigation is needed to determine whether or not our findings are valid in various kinds of humors. The objective measurement of such expectations of humorous events may be extended so that it can be used to evaluate the performance of humorous movies. Movie producers and comedians would be able to improve their products and performances if they had a means to improve movie scenes in which viewers expected an upcoming humor event but could not reach the level of laughter. The ability to extract mental states during movie viewing would also lead to more convincing critiques of movie content; so far, movies are evaluated mainly on the basis of opinions of experienced professionals. Further development of a means to extract mental states during movie viewing may eventually allow evaluations not only of comedies but also of dramatic movies that should be rated by affective perspectives.
